# Effectiveness and efficiency of a practice accreditation program on cardiovascular risk management in primary care: study protocol of a clustered randomized trial

**DOI:** 10.1186/1748-5908-7-94

**Published:** 2012-10-04

**Authors:** Elvira Nouwens, Jan Van Lieshout, Eddy Adang, Margriet Bouma, Jozé Braspenning, Michel Wensing

**Affiliations:** 1 Scientific Institute for Quality of Healthcare (IQ healthcare), Radboud University Nijmegen Medical Centre, PO Box 9101/Geert Grooteplein 21, 114 IQ healthcare, Nijmegen, HB 6500, The Netherlands; 2 Department of Epidemiology, Biostatistics and HTA, Radboud University Nijmegen Medical Centre, PO Box 9101/Geert Grooteplein 21, 113 EBH, Nijmegen, HB 6500, The Netherlands; 3 Dutch College of General Practitioners, Domus Medica, Mercatorlaan 1200, Utrecht, BL, 3528, The Netherlands

**Keywords:** Institutional accreditation, Professional certification, Primary care, Cardiovascular illness

## Abstract

**Background:**

Cardiovascular risk management is largely provided in primary healthcare, but not all patients with established cardiovascular diseases receive preventive treatment as recommended. Accreditation of healthcare organizations has been introduced across the world with a range of aims, including the improvement of clinical processes and outcomes. The Dutch College of General Practitioners has launched a program for accreditation of primary care practices, which focuses on chronic illness care. This study aims to determine the effectiveness and efficiency of a practice accreditation program, focusing on patients with established cardiovascular diseases.

**Methods/design:**

We have planned a two-arm cluster randomized trial with a block design. Seventy primary care practices will be recruited from those who volunteer to participate in the practice accreditation program. Primary care practices will be the unit of randomization. A computer list of random numbers will be generated by an independent statistician. The intervention group (n = 35 practices) will be instructed to focus improvement on cardiovascular risk management. The control group will be instructed to focus improvement on other domains in the first year of the program. Baseline and follow-up measurements at 12 months after receiving the accreditation certificate are based on a standardized version of the audit in the practice accreditation program. Primary outcomes include controlled blood pressure, serum cholesterol, and prescription of recommended preventive medication. Secondary outcomes are 15 process indicators and two outcome indicators of cardiovascular risk management, self-reported achievement of improvement goals and perceived unintended consequences. The intention to treat analysis is statistically powered to detect a difference of 10% on primary outcomes. The economic evaluation aims to determine the efficiency of the program and investigates the relationship between costs, performance indicators, and accreditation.

**Discussion:**

It is important to gain more information about the effectiveness and efficiency of the practice accreditation program to assess if participation is worthwhile regarding the quality of cardiovascular risk management. The results of this study will help to develop the practice accreditation program for primary care practices.

**Trial registration:**

This cluster randomized trial is registered at ClinicalTrials.gov nr NCT00791362

## Background

Cardiovascular diseases (CVD) remain an important cause of mortality and morbidity worldwide. In public health and in primary care, many efforts have been made to prevent CVD [1,2]. Although cardiovascular care has improved in recent years [3], a substantial number of individuals receive suboptimal cardiovascular risk management (CVRM) and do not attain the lifestyle, risk factor, and therapeutic targets that are recommended [4,5]. A range of interventions to improve healthcare delivery is available [6-8]. In recent years, programs have been developed for performance indicators, accreditation, pay-for-performance, and public reporting [9]. These approaches make use of market forces and pressure for accountability, but research evidence on effectiveness and efficiency is limited [9-11].

The slow improvement of cardiovascular primary care may be caused by the one-off and condition-specific character of many improvement activities (*e.g.*, a continuing education session or audit without follow-up). To enhance continuous improvement in primary care in the Netherlands, the Dutch College of General Practitioners (DCGP) initiated in 2005 a nationwide comprehensive practice accreditation program for primary care practices. This program consists of a systematic audit on the basis of validated performance indicators for diabetes mellitus, CVRM, asthma, chronic obstructive pulmonary disease (COPD), practice organization, patient experience, educational feedback to practices, the requirement to develop structured improvement plans, and a check on the implementation of these plans after one year. If the procedure is performed, primary care practices receive a certificate that provides accreditation for a time period of one year. While accreditation serves a range of purposes, improvement of professional performance and practice organization are prominent among these [12-14]. While the impact of audit and feedback is mixed and moderate [15], it is unknown what the added value of the accreditation procedure is. A study of practice accreditation in German primary care practices showed that it improved aspects of practice organization, but this study did not measure or assess impact on clinical processes or outcomes [16]. Given the resources invested in accreditation schemes and the high expectations, an evaluation of the impact on quality and outcomes of care is needed.

A substantial number of performance indicators used in the practice accreditation program is related to CVD. These indicators are derived from the completely revised guideline on CVRM that was published by the DCGP late 2005 [17]. The new set of guidelines on CVRM describes the clinical interventions to be implemented in patient care in this project. They contain important changes in recommendations, such as different cut-off levels (*e.g.*, LDL-cholesterol <2.5 mmol/l and systolic blood pressure <140 mmHg). The DCGP has developed a number of products and activities to implement these guidelines, including a national kick-off conference for general practitioners (GPs) in December 2005, and a supportive package (‘kwaliteitskoffer’) consisting of educational materials and software for assessment of cardiovascular risk. The practice accreditation program is an important approach to improve primary care, but controlled evaluations of its effect have not yet been done.

### Aims and objectives

The overall aims of the study are to determine the effectiveness and efficiency of the practice accreditation program in primary care, focused on its effect on CVRM.

Key objectives are:

1. To determine the effectiveness of the program on primary performance indicators for CVRM by comparing practices in the accreditation program that focus their improvement plans on CVRM to practices in the accreditation program that focus their improvement plans on other domains of chronic care. Primary outcomes are documented controlled blood pressure, serum cholesterol, and prescription of recommended preventive medication (effect evaluation).

2. To determine the potential effect of the program on other indicators for CVRM, self-reported goal attainment in the intervention group, and unintended consequences. Secondary outcomes are all other indicators for CVRM, self-reported goal attainment in the intervention group, and unintended consequences (effect evaluation).

3. To determine the economic efficiency of the program in the observed period regarding the primary outcomes.

4. To explore what factors and mechanisms are associated with change (or absence of change) of performance in CVRM.

## Methods/design

### Trial design

The study design is a cluster randomized controlled trial with a block design, considering primary care practices as units of clustering.

### Ethical approval and informed consent

The Medical Ethics committee Arnhem-Nijmegen waived approval for this trial. At follow-up practices will send a letter of invitation and informed consent to 100 patients with established CVD. Patients return their letter with informed consent to Radboud University Nijmegen Medical Centre with permission for audit of their medical records. The privacy of the participating patients will be protected, and all data will be coded and processed anonymously. For the baseline-measurement, mandatory information on indicators for patients with established CVD will be used, collected by practices on behalf of the practice accreditation program.

### Participants

#### Primary care practices

Seventy primary care practices will be recruited from practices in the Netherlands who voluntarily apply to start the practice accreditation program. An invitation letter for the study will be sent as part of the instruction manual for the program. Practices with a clear preference for a specific improvement plan will be excluded from participation in the study while participating practices will be randomized to a group which starts with an improvement plan on CVRM or to a group that does not. This also accounts for practices that participate in regional programs for enhancing disease management.

#### Patients

The study focuses on patients with established CVD. This includes patients with angina pectoris, acute myocardial infarction in their medical history, other chronic ischemic heart diseases, transient ischemic attack (TIA), ischemic stroke, peripheral arterial disease, and aneurysma aortae. Selection of patients out of electronic medical records with these conditions will be based on corresponding diagnostic International Classification of Primary Care codes (ICPC-codes K74, K75, K76, K89, K90.3, K92.1 and K99.1), a worldwide system to label conditions in primary care [18].

#### Randomization

General practices will be the unit of randomization. A computer list of random numbers will be generated by an independent statistician and then used to randomly allocate practices to equally sized intervention group or control group. This will be done in a randomized block design in blocks of four practices in order of enrolment. We assume that improvement activities in the control group will not influence cardiovascular care.

#### The practice accreditation program

The practice accreditation program is an existing procedure provided since 2005 by an independent body (NPA) that has a license to use the accreditation procedure developed by the DCGP. The DCGP remains responsible for the content and further development of the procedure; it will be responsible for adequate delivery of the practice accreditation program in this study. The practice accreditation program comprises, firstly, of a comprehensive audit (using validated performance indicators) and written feedback to the practice, which covers a range of clinical domains (mainly chronic diseases), practice management, and patient experiences. The feedback, which consists of a comparison with benchmarks of other primary care practices, is discussed with a non-physician observer in a feedback consultation and helps to identify substandard performance. The second obligatory component, the planning of improvements in the practice according to the principles of quality management, is based on this feedback. The practice team is supported by a trained non-physician consultant. Practices that perform the procedure as planned are all accredited, so accreditation does not imply that a certain minimum score has been obtained (the latter is usually labeled certification). In the practice accreditation program, validated instruments are used: the Visit Instrument to asses Practice management (*Visitatie Instrument Praktijkvoering*, VIP) [19], clinical performance, and Europep [20]. Practices in the program receive a reimbursement of some insurance companies consisting of a bonus per patient per year. Furthermore they receive a certification for the time period of one year that demonstrates (to the public) their involvement in continuous quality improvement. Every year the practice will be audited, and every year new improvement plans have to be formulated that have to be approved by the auditor.

#### Intervention group

The intervention starts with volunteering for the practice accreditation program. After enrolment for the study, practices will be contacted by telephone for further explanation of the study protocol and to schedule the data collection. After data is collected, practices are randomized. Practices allocated to the intervention group are instructed to focus their improvement plans on cardiovascular diseases in the first year of the program.

#### Control group

The control group also starts with volunteering for the practice accreditation program and follows the same routine as described for the intervention group. Practices allocated to the control group are instructed to focus their improvement plans on other domains than cardiovascular disease and diabetes mellitus (they may target CVD later after the study period of one year). They are instructed to focus their improvement plans on other clinical areas than CVD or diabetes.

Both intervention and control group receive feedback on CVD indicators as part of the normal practice accreditation program. Practices in the intervention group are instructed to set targets related to process and outcomes of cardiovascular care (and not just focused on improvement of registration of cardiovascular disease in the medical record system). All practices will receive a minimum of four hours of support by outreach consultants for no cost, which is available in all regions. Also, the practices are provided with examples of improvement plans, which saves time and would make study participation more attractive.

#### Measurement procedures

**Figure 1:**
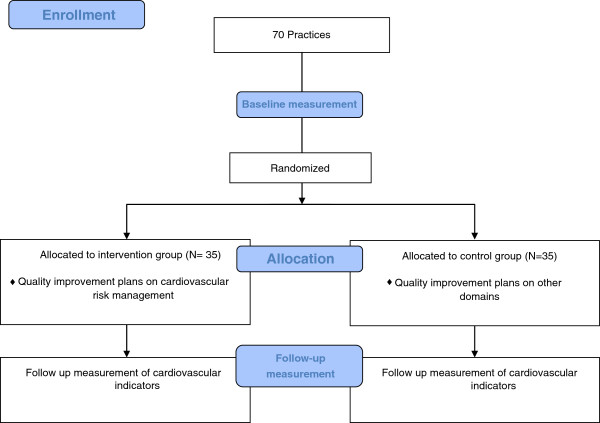
Flow Diagram.

In each practice measurements are done at baseline and at follow-up (Figure 1). At baseline, medical records of 40 patients with established CVD are audited as part of the clinical performance measurements in the practice accreditation program. Data on performance indicators of CVRM as included in the practice accreditation program will be used for the analysis. At follow up, the following measurement methods will be used: medical record audit based on the same indicators of CVRM as in the baseline measurement, patient questionnaires, and a semi-structured interview for a contact person in each practice. Data will be collected consistently as this is done by two persons with similar training.

#### Measures of effectiveness

The effect evaluation aims to determine the effectiveness of the program on primary performance indicators for CVRM and to determine the potential impact of the program on other indicators for CVRM, self-reported goal attainment in the intervention group, and unintended consequences.

Primary outcomes have been selected from the 20 quality indicators for established CVD [21], which were developed by DCGP (Table 1), and are:

1. The percentage of patients in the practice with known established CVD who have systolic blood pressure below 140 mmHg.

2. The percentage of patients in the practice with known established CVD who have LDL cholesterol below 2.5 mmol/l.

3. The percentage of patients in the practice with known established CVD with a record that aspirin, an alternative anti-platelet therapy, or an anticoagulant has been prescribed.

**Table 1:** Indicators for cardiovascular risk management

**Type of indicator**	
Process	Smoking status
Outcome	Patient is a smoker
Process	Stop smoking advice
Process	BMI measured
Outcome	BMI < 25 kg/m^2^
Process	Influenza vaccination
Process	Exercise control
Process	Systolic blood pressure measured
Outcome	Systolic blood pressure < 140 mmHg^1^
Process	LDL cholesterol measured
Outcome	LDL cholesterol <2.5 mmol/L^1^
Process	Advice physical activity
Process	Diet control
Process	Counseling about diet
Process	Registration of alcohol intake
Process	Patients with LDL cholesterol ≥2.5 mmol/L with statin prescription
Process	Waist circumference measured
Process	Prescription antiplatelet drugs^1^
Process	Fasting glucose measured
Process	Comprehensive risk assessment *

*Positive score when there is a record of: blood pressure, BMI, waist circumference, fasting glucose measurement, LDL cholesterol measurement, smoking behavior, alcohol intake, advice and control of diet and physical exercise in the past 12 months.

^1^Primary outcome.

Data concerning indicators are extracted from medical records and will be available at patient level so that linkage to other measures (resource use, patient characteristics) can be made at patient level.

Secondary outcomes consist of the 17 remaining indicators are used and include: measurement of systolic blood pressure, measurement of LDL-cholesterol, prescription of statin, smoking status, stop smoking advice, measurement of body mass index (BMI), BMI <25 kg/m^2^, measurement of waist circumference, fasting glucose measurement, influenza vaccination, registration of alcohol intake, control and advice for exercise and diet, and comprehensive risk assessment (Table 1). Furthermore, secondary outcomes are measured in interviews with the contact person of the practice and contain perceived goal attainment regarding the improvement plans, which is measured on a likert scale, and unintended consequences as result of participating in the practice accreditation program.

#### Measures of costs

In follow-up measurements items of use of healthcare will be extracted from the medical records with a retrospective three-month observation period. These items include number of contacts in the practice (face to face, telephone, email), use of various types of cardiovascular medication, use of hospital care or other care providers for cardiovascular diagnosis or therapy. Additional information will be collected with patient questionnaires, particularly on other healthcare use (*e.g.*, home care) and productivity losses, using a one-month retrospective observation period. Also, at follow-up in both groups, time and other resources of practice teams spent on quality improvement in the total period of 18 months will be documented. Data-collection on performance indicators will be done in the follow-up measurement by medical record audit.

#### Other measures

1. Exposure to other quality improvement activities: Both study groups report on their exposure to relevant professional education and practice improvement activities (*e.g.*, training for practice nurses). This will be measured in semi-structured telephone interviews.

2. Potential confounders: At follow up, potential confounders will be measured. These include patient characteristics, particularly patient age, gender and multi-morbidity. Furthermore, data on practice characteristics will be collected. These include practice size, physician workload, volume of assistance in the practice, delegation of medical tasks to assistants, and involvement of practice nurses in chronic care. These practice characteristics have shown to be associated with better chronic disease management in Dutch primary care practices [22].

3. Patient reported outcomes: At follow up, patients receive questionnaires that include items on demographic characteristics, labor activities and healthcare use. Furthermore the EQ-5D (five items and VAS scale) will be added to measure health outcome [23]. To measure chronic care delivery, the Patient Assessment of Chronic Illness Care (PACIC) will be used [24]. Questionnaires for physical exercise (RAPA, nine items) [25], and the Treatment Self-Regulation Questionnaire (TSRQ) [26] to measure the motivation for being physically active will be included.

#### Statistical methods

The study groups will be compared at baseline regarding known determinants of cardiovascular care and its improvement. These include patient factors [27,28] (*e.g.*, age, multi-morbidity, ethnicity at practice level) and practice characteristics [22,29] (*e.g.*, availability of nurses, delegation of medical tasks to assistance, practice size). Only factors emerging from previous research are considered to avoid overcorrection in the primary analysis. A logistic regression model will be constructed for each outcome to analyze these outcomes in relation to group (intervention, control) and measurement moment (baseline, follow-up). Identified differences between the groups at baseline will be included in this analysis. Random coefficients will be included to allow for the clustering of data within practices. Each of the secondary outcomes (clinical and organizational indicators) will also be analyzed in this way. Finally, if an internally consistent scale can be constructed (reflected by high reliability coefficients of the combined score), we will develop an aggregated measure of outcome, and use this in a similar random coefficients linear regression analysis.

To identify the effectiveness of this program on attainment of practice-defined goals and its perceived unintended consequences, the second key objective, a descriptive analysis will be performed aimed at determining what proportion of self-defined goals for improvement was achieved by the practices and straightforward listing of the GP views on unintended consequences of the practice accreditation program.

#### Economic analysis

The economic analysis, the third key objective, aims to determine the efficiency of the program in the observed period regarding the primary outcomes. The economic evaluation also investigates the relationship between costs, performance indicators, and accreditation. The economic evaluation provides incremental cost-effectiveness ratios: incremental cost per percentage patients gained with systolic blood pressure below 140 mmHg; incremental cost per percentage patients gained with LDL cholesterol <2.5 mmol/l; incremental cost per percentage patient gained with aspirin, an alternative anti-platelet therapy or an anti-coagulant.

For the economic analysis, costs analyses will be based on the competing health production processes respectively, including and excluding resources attributed to accreditation. Specific unit-costs include, for example medical care (contacts in primary care practice, tests, treatments, etc.) and improvement related costs (accreditation tariff, time for audit, planning and implementing improvement, exposure to other relevant quality improvement, etc.). Units of resources are monetary valued on the basis of prevailing Dutch guidelines [30] or national CVZ tariffs. The analysis aims to provide incremental cost-effectiveness ratios (ICERs). The ICERs will be computed, and uncertainty will be determined using the bootstrap method to account for skewness in the underlying parameter distributions. Uncertainty will be presented in a Bayesian fashion using cost-effectiveness acceptability curves (CEAC’s) that are able to evaluate efficiency by using different thresholds for the ICER (varying the willingness to pay for a percentage patients gained for each of the primary outcomes). Uncertainty in deterministic parameters will be examined with sensitivity analysis based on the range of extremes.

#### Process evaluation

The process evaluation, the fourth key objective, aims to explore what factors and mechanisms are associated with change (or absence of change) of performance on CVRM indicators. Semi-structured telephone interviews will be held with primary care physician or the quality coordinator of the practice after data collection for follow-up measurement is concluded. Topics of this interview are: practice characteristics; feedback reports; composing improvement plans; and reasons to participate in and experiences with the practice accreditation program. We want to explore if specific elements of the practice accreditation program are the cause of change in CVRM. All interviews will be conducted by the same person and will be audio-recorded. Interviews will be transcribed verbatim. Two researchers will independently review the transcripts. Data analysis will be done according to the framework approach [31]. Topics in the interviews will be used as coding frame. Software package Atlas ti. will be applied to analyze qualitative data. The primary analysis of the interview data aims to identify determinants of (change of) practice as perceived by participants.

#### Sample size

In the practices in the practice accreditation program up to 2006 (n = 139), the following median values at practice level were found on indicators referring to patients with CVD: 53% for acceptable blood pressure levels; 36% for acceptable cholesterol levels; and 38% for use of anticoagulents (unpublished data at IQ healthcare, 2005 and 2006). These data suggest that the current scores on the primary outcomes are in the range of 35% to 55%, which imply that substantial improvement is possible in many practices. The proposed study has been powered to detect a difference of 10% (from 55% to 65%), not yet taking control for baseline values into account because of uncertainty regarding the correlation between baseline and follow-up measures.

We expected that the accreditation and improvement program will have an effect of 5% to 10% absolute change, which is the median value of effect sizes in a comprehensive review of 235 studies on quality improvement [14]. Other assumptions were a power = 0.80, alpha = 0.05, and ICC = 0.05. Given the sample of 30 patients per practice per indicator, we aimed to include 31 practices in each group. Allowing for dropout, we aim to include 35 practices in each group (n = 70 practices in total). This number is feasible, given the recruitment rate for the accreditation in 2006.

#### Time frame of the study

The study is planned from September 2008 until September 2012. In months 1 to 18, practices are recruited and included in the project and go through the accreditation procedure. The baseline data collection will take place in these months. During months 3 to 42, practices (in the intervention group) work on improving their management of CVD, practices in the control group on improvements in other areas. In months 18 to 42, follow-up measurements in intervention and control practices are planned. During months 43 to 48, data will be analyzed and reported.

## Discussion

The sample of participating primary care practices in this study is composed of volunteers for the practice accreditation program and therefore not nationally representative for primary care practices in the country. This reflects current practice, in which practice accreditation is a voluntary activity. It implies that study results cannot be generalized to the (currently hypothetical) situation of obligatory accreditation. Furthermore, we only collect data on CVRM; therefore, we cannot make statements about the effects of the program on other chronic illnesses.

Because both intervention and control groups start with accreditation, this project cannot pick up non-specific effects of the practice accreditation program. For example, we expect that practices prepare for accreditation by improving their practice (*e.g.*, involve a practice nurse). We intend to compare the groups with other, independent samples of practices that provide data on cardiovascular care to get an impression of the representativeness of our sample of practices.

With the results of this study, we hope to make a contribution with regard to further development and adjustment of the practice accreditation program. Previous research [32] has shown that the program is time-consuming for participating practices. Furthermore, it costs a considerable amount of money to participate in the program. It is important to gain more information about the effectiveness and efficiency of the program to assess if participation is worthwhile regarding the quality of CVRM. With these results, stakeholders can make policy and management decisions with regard to the use of the program.

No data cleaning or analysis has occurred prior to submission of the manuscript.

## Abbreviations

CVD: Cardiovascular disease; CVRM: Cardiovascular risk management; DCGP: Dutch College of General Practitioners; COPD: Chronic obstructive pulmonary disease; GP: General Practitioners; LDL-cholesterol: Low Density Lipoprotein-cholesterol; TIA: Transient Ischemic Attack; VIP: The Visit Instrument to asses Practice management (Visitatie Instrument Praktijkvoering); ICPC: International Classification of Primary Care; PACIC: Patient Assessment of Chronic Illness Care; TSRQ: Treatment Self-Regulation Questionnaire; ICER: Incremental cost effectiveness ratio; CEAC: Cost-effectiveness acceptability curve.

## Competing interests

MW is Co-Editor in Chief of Implementation Science; all decisions on this paper were made by another editor. EN, JL, EA declare that they have no competing interests. JB is involved in development of the practice accreditation program and receives funding for this from the Dutch College of General Practitioners. MB is an employee at the Dutch College of General Practitioners and is involved with the practice accreditation program.

## Authors’ contributions

EN is involved in developing instruments, data-collection, analysis, and reporting aspects of the trial. MW, the project leader, is involved in all aspects of the study. JvL and JB are involved in the design of the study, the analysis, and the reporting. MB is involved in the design and reporting of the study. EA will evaluate the economics. All authors read and approved the final manuscript.
